# Report on the 2014 UKONS Annual Conference Belfast, 14–15 November 2014: focus on living with and beyond cancer, patient information and support, and innovations in treatment and care

**DOI:** 10.3332/ecancer.2015.509

**Published:** 2015-02-10

**Authors:** Richard Henry, Lallita Carballo, Gillian Knight, Elaine Lennan, Helen Roe

**Affiliations:** 1 Queens University, University Road, Belfast BT7 1NN, Northern Ireland, UK; 2 University College London Hospitals (UCLH), 235 Euston Road, Fitzrovia, London NW1 2BU, UK; 3 South Wales Cancer Network, Glanrhyd Hospital, Pen-y-Fai, Bridgend CF31 4LJ, Wales, UK; 4 University Hospital, Southampton, Hampshire SO16 6YD, UK; 5 North Cumbria University Hospitals, NHS Trust, Carlisle, CA2 7HY, UK

**Keywords:** oncology nursing, United Kingdom Oncology Nursing Society (UKONS), innovations in treatment and care, patient information and support, living with and beyond cancer

## Abstract

The UK Oncology Nursing Society’s (UKONS) annual conference focused on three major themes. These were ‘Living With and Beyond Cancer’, ‘Patient Information and Support’, and ‘Innovations in Treatment and Care’. It featured a wide range of presentations, industry satellites, exhibitions, poster discussions. and workshops. Presenters ranged from those eminent in their particular field to those gracing the speaker’s podium for the first time. The rich variety of presentations covered policy, cancer trends, clinical developments, care initiatives, personal development, and advances in practice. There was a strong emphasis on skills, knowledge, values, and attitudes, with the most junior and novice nurses mixing with experienced and highly esteemed practitioners.

## Introduction

The UKONS seeks to promote excellence in nursing care and the management of all those directly and indirectly affected by cancer. It seeks to achieve this by encouraging and facilitating the personal and professional development of nurses engaged in cancer care throughout the UK and overseas through education, research, and policy. Additionally, the society influences and advises on cancer policy, clinical practice, and service delivery nationally and internationally.

More than 260 delegates from the UK and Ireland attended the 2014 UKONS Annual Conference in the Europa Hotel in Belfast. This year’s conference focused on ‘Living With and Beyond Cancer’, ‘Patient Information and Support’, and ‘Innovations in Treatment and Care’, and featured a wide range of presentations, industry satellites, exhibitions, poster discussions, and workshops. Presentation and workshop slides can be found on the UKONS website (http://www.ukons.org/conference).

The conference was opened by Jim Wells MLA, the Northern Ireland Minister for Health, Social Services, and Public Safety. The Minister noted that people with cancer are living longer than ever before and highlighted the continuing and increasing importance of the nurses in the treatment and care of these patients.

## Cancer survivorship and the Recovery Package

More people are living for longer periods with and beyond cancer. This theme was taken up by Professor Jane Mayer, Chief Medical Officer at Macmillan Cancer Support, in her presentation titled ‘Living with and Beyond Cancer—The Recovery Package’. Professor Mayer highlighted the issue of the unmet needs of cancer patients and their impact on survivorship. She explained that unmet needs were particularly apparent in the gaps between care settings as a patient moves through the different stages of their disease. The Recovery Package offers a means of identifying and addressing these needs and thereby enhancing the lives of those living with and beyond cancer. However, when considering challenge of cancer survival, Professor Mayer noted that mortality rates differ among cancers, as do patterns of care and relapse, comorbidities, and multidisciplinary team interactions—all of which need to be considered.

The Recovery Package was developed and tested by the National Cancer Survivorship Initiative in 2008 (NCSI, 2012) [2]. It makes use of a series of interventions (assessment and care planning, treatment summary and cancer care review, and health and wellbeing events) which are integral to supporting self-management. Professor Mayer noted that nurses are well placed to identify a range of needs and can positively impact the quality of life of those living with and beyond cancer.

Professor Mayer specifically referred to Northern Ireland’s experience of the Recovery Package in breast cancer services. In this it was found that most patients felt they ‘received the medical support needed’, many felt less supported with the physical, emotional, practical, and financial impact of their disease. The introduction of the Recovery Package has elicited very positive patient feedback and instigation of pathway redesign across Northern Ireland.

In a similar vein, Professor Josephine Hegarty, Acting Dean of the School of Medicine and Health, University College Cork presented her results on the experiences of individuals who have lived with, through and beyond cancer. This complemented Professor Mayer’s address by highlighting some of the needs experienced by people with cancer and how these needs can vary by stage, patient age, cancer type, and presence/absence of comorbidities. Professor Hegarty identified trends in patient needs, demonstrating that the presence of a problem does not always correlate with the need for assistance. Professor Hegarty concluded that understanding the long-term effects of cancer can help health and social services to be better prepared to assist those surviving with the disease. To support this, she highlighted the need for effective communication with patients and their families.

## Inequalities in cancer care

Mark Lawler, Professor of Translational Cancer Genomics at Queen’s University Belfast, addressed treatment inequalities and ageism in cancer care. Referring to the European Cancer Patient’s Bill of Rights (Lawler *et al*, 2014) [1], Professor Lawler argued that it is the right of every European citizen to receive accurate information about their disease and options. They should be proactively involved in decisions about their treatment, have equal and timely access to specialised care, and receive care underpinned by research in sustainable health systems designed to ensure optimum outcomes. Professor Lawler highlighted some of the inequalities in care between—and within— England, Scotland, and Northern Ireland. Information was a key point, suggested Professor Lawler, for without information people could not become ‘active participants in care’ but remain ‘passive recipients’. Challenging the common use of chronological age to determine treatment options, Professor Lawler highlighted the growing body of evidence which suggests that older patients are less likely to receive he treatment they need based on their age.

## Supporting staff to care: connecting staff and patient experience

Professor Jill Maben, Director of the National Nursing Research Unit at King’s College London, highlighted the importance of care, compassion, and ideals among nursing staff. Professor Maben shared research findings on how the ideals of recently qualified nurses often change as they are exposed to realities of work. These changes include the erosion of nursing ideals and values. She identified three groups of nurses, those sustained idealists (those who held onto their ideals), compromised idealists (those who adjusted their ideals to fit reality), and crushed idealists (those who lost their ideals). She suggested, that within a year, most nurses were among the crushed or the compromised idealist groups. When exploring the reasons behind this negative response Professor Maben identified time pressures, role constraints, lack of support, staff shortages/work overload, and task-orientated rather than patient-focussed care. Professor Maben then introduced a model for a positive environment of care.

## Families’ discussion of hereditary cancer risk: implications for nursing care

Given that 5–10% of diagnosed cancers have a hereditary element, there is a growing need for improved healthcare service-to-patient and patient-to-family communication according to Alison Metcalfe, Professor of Health Care Research and Associate Dean for Research at King’s College London. She discussed current practice in this regard and shared the findings of more than 50 interviews with parents, children, and young people.

Cancer genetic counsellors discuss the implications of genetic tests, review family histories, and inform their clients of the likelihood of the presence of an inherited gene mutation. Whether the test results are positive, negative, or ambiguous, those receiving the news may experience feelings of guilt, anxiety, or depression. The management of these consequences, as well as how best to communicate risk to family members, is beginning to find a place in mainstream cancer nursing.

Professor Metcalfe found that people with cancer need support in talking to their families. This can be particularly acute as there are a range of barriers which inhibit this. These barriers might be emotional in that parents can feel the need to protect their children. Poor communication, lack of information and support together with difficult family relationship are among the other reasons for these difficulties.

## Workshops

### Innovations in treatment and care workshop

Chairs: Gillian Knight, Elaine Lennan, and Wendy Anderson, UKONS Board Members

#### DAY 1

**Hepatocellular carcinoma (HCC) in immigrant population: impact of viral status**

Sofi Dhanaraj, Liver Clinical Nurse Specialist, Queen Elizabeth Hospital, University Hospitals Birmingham, NHS Foundation Trust

Sofi Dhanaraj referred to her research into HCC in the immigrant population and the impact of a viral status. The incidence and mortality rates of HCC have been rising in the UK over the last 30 years. Little is known about the impact of the disease distribution among specific ethnic groups. This research found that immigrant populations may be at increased risk of HCC because of their viral status. It needs to be recognised that this risk group should be screened for HBV and HCV infection so that early diagnosis and appropriate screening may allow early detection of HCC and a consequent improvement in survival. The data and results provide evidence for the need for continuing research based on the distinguishable patterns in aetiology and HCC incidence among immigrants.

**Physiotherapy-led and exercise instructor-led physical activity programmes for cancer patients: Is there a need for a stratified approach to referral and signposting?**

Catherine Neck, Macmillan Cancer Rehabilitation Lead, Southmead Hospital, North Bristol, NHS Trust

This paper presented outcomes from two physiotherapy-led and five exercise instructor-led programmes that have been set up across the Bristol region. All programmes offer a circuit-style programme, some of the leisure centres also offer gym programmes. All patients are assessed before being given individualised programme. On completion they are signposted to ongoing exercise activities that they can continue with. Physical, psychological, and functional outcome measures are collected as well as a programme evaluation on completion. The results here highlighted that all programmes demonstrated positive results in all domains, and that there is a role for both exercise instructor and physiotherapy-led programmes for people living with and beyond a cancer diagnosis.

**Evaluation of a polymeric membrane dressing in the management of radiotherapy skin induced reactions in head and neck cancer patients**

Audrey Scott, Macmillan Head and Neck Clinical Nurse Specialist, Mount Vernon Hospital, The Hillington Hospitals, NHS Foundation Trust

This paper presented findings from an evaluation of use of a polymeric membrane dressing used with those head and neck cancer patients who experience moderate to severe skin reactions following radiotherapy. Patients were provided with a free text diary to log their pain using a numerical scale and Wong and Baker FACES scale. Patient diaries provided valuable data on common themes including increased sleeping hours, dramatic reduction in pain during wear time of the dressing, and increased healing rates. Practice has now changed and the use of the advanced wound dressing has improved the quality of life in these vulnerable patients. Patient participation was the key, and the diaries provided invaluable feedback.

#### DAY 2

The workshop offered examples of innovations in treatment and care from Wales. The workshop was divided into two parts with four presentations in the first part and an interactive introduction to tools and practice models in the second.

Rosie Roberts, Chemotherapy Specialist Nurse from Velindre Cancer Centre, Cardiff offered an insight into two chemotherapy related projects. The first involved a review of chemotherapy helpline calls that were about nausea and vomiting. Many calls were from breast cancer patients who were receiving flourouracil, epirubic, and cyclophosphamide (FEC). This led to a review of the antiemetic policy in the unit. Chemotherapy patient education was the focus of the second development, and it involved group chemotherapy patient education sessions.

Rhianydd Jones, a research link nurse and Kay Wilson, an early phase trials nurse from the Cancer Tertiary Early Phase Clinical Trials Unit in Cardiff presented on the development of a clinical trials treatment unit. This offered an exploration of the practicalities around scoping, benchmarking, securing funding, a designated treatment unit, and the team establishment.

David Williams, Information and Improvement Lead from Public Health Wales discussed measurement taken based on evidence change. The Welsh Improving Quality Together, a learning programme on quality improvement was outlined highlighting that this has now become the backbone for quality improvement in NHS Wales. It is integrated into learning and development programmes in all Health Boards, Trusts and Universities, and has been put into practice.

Michelle Pengelly, Specialist Nurse in Supportive Care gave an insight into advancements in care through patient stories. Michelle commenced by asking ‘Can one cancer patients story change and influence practice?’, and then explored patients and significant other stories as a quality improvement methodology.

The second part of the session was a workshop interactive session which focused on tools, techniques. and enablers to support local innovations. This invited and gave those attending the workshop an opportunity to explore a number of change models and consider them in the context of projects or changes needed in their own organisations. The practical process of how to get started, whom to collaborate with, how to measure impact, and other enabling tools were all explored in this session.

## Patient information and support: poster discussion session and workshop

Chairs: Helen Roe and Lallita Carballo, UKONS Board members

**Day 1:** The first day offered a mixture of both novice and experienced speakers presenting on issues relating to the information needs of patients receiving trial drug therapy, a very underdeveloped area of information support. Professor Emma Ream presented on the results of a systematic review of the barriers to early presentation, and diagnosis with breast cancer amongst black women.

**Day 2: Patient information and support workshop**

This workshop aimed to provide participants with current and up-to-date evidence on why patients and their families need to access supportive care services drawing on the research on the unmet needs of cancer patients.

Dr Jo Armes, Supportive Cancer Care Group, Florence Nightingale Faculty of Nursing and Midwifery, King’s College London, presented on the unmet supportive care needs of patients and families. She made the case for ensuring that practitioners address supportive care needs early on in the cancer pathway to prevent significant unmet need post treatment and beyond.

Dr Reg Race from Quality Health presented the most recent data from the 2014 National Cancer Patient Experience Survey (NCPES). He focused on those questions that related to support and information. This seemed to demonstrate that the most significant antecedent to a positive patient experience was the involvement of a clinical nurse specialist.

The afternoon session focussed on exploring current innovations and initiatives aimed at improving and addressing supportive care needs. Contributors included Danny Bell from Macmillan Cancer Support (who is leading on a range of national projects to support patients during treatment and recovery) and Karen Verrill from Maggie’s in Newcastle who highlighted this particular model of supportive care in action. Ann Finn and Emma King presented their work in Northern Ireland which involves a sustainable means of ensuring that practitioners have access to high quality advanced communications skills training. Finally, Hilary Plant presented work on a survey to evaluate the experience of patients using a ‘drop in’ service within a supportive care centre at UCLH.

## Living with and beyond cancer

Chairs: Natalie Doyle, UKONS Past President, and Cathy Hughes, UKONS Board Member

### 

#### DAY 1

Promoting the health and wellbeing of men with testicular cancer through information and support

Wendy McPhee, Staff Nurse, Chemotherapy Day Unit, Belfast Health and Social Care Trust (Formerly Macmillan Uro-Oncology Project Nurse)

Men with a diagnosis of testicular cancer can experience complex physical, psychological, and social problems. Wendy McPhee discussed a series of health and wellbeing events which were designed to address these issues. These events help patients to manage their own health and take an active role in sustaining recovery; they promote the health and wellbeing of young men with testicular cancer and improve patient’s and carer’s understanding of the treatments available and their associated side effects. The events introduce the concept of survivorship, and it also raises awareness of the services that are available. The events have almost achieved a 100% patient, carer and healthcare professional satisfaction rating.

What are the experiences of survivors of teenage and young adult cancer when receiving a copy of their survivorship care plan?

Tanya Urquhart, Macmillan Clinical Nurse Specialist, Sheffield Children’s, NHS Foundation Trust

Malignant disease is the second most common cause of death in teenagers and young adults. Given their very unique and diverse needs, these patients have been described as ‘the lost tribe’. Tanya Urquhart researched the reaction of these patients to receiving their survivorship care plans (SCPs). Using semi-structured, digitally recorded, face-to-face interviews, she found that patients used their SCPs to support them in moving on, as a communication tool, and to help them reflect on and recall previous information given to them. Overall, the evidence suggests that SCPs can be overwhelming but are invaluable. Their routine use is recommended.

End of treatment consultations for women with breast cancer

Jo Armes, Supportive Cancer Care Group, Florence Nightingale Faculty of Nursing and Midwifery, King’s College London

There is growing evidence that unmet needs after treatment do exist. To improve the care and support offered to women with early breast cancer a nurse-led End of Treatment Consultation was conducted. This consisted of 45-minute appointments taking place four to six weeks after the completion of the main phase of treatment. Holistic assessments of physical, psychological, informational, and social needs were carried out, and if any unmet needs were identified, patients were referred to ensure ongoing support. Patients appreciated these consultations, which helped to provide the required information, support, reassurance, and navigation of healthcare systems at a time of vulnerability.

#### DAY 2

Facilitated by Dr Tim Anstiss, this interactive workshop was designed to develop the confidence and skills of nurses in helping patients discover and strengthen their motivation and readiness to make beneficial lifestyle changes.

TimAnstiss introduced the workshop by highlighting some of the major benefits of changing the way healthcare professionals give information to patients and elicit change through the use of a series of exercises and videos. He reinforced the need to stay away from attempting to resolve the individual’s issues by informing them what they should/should not be doing. Rather, the person should be encouraged to realise and vocalise these issues themselves, and then come up with ways to resolve them. Dr Anstiss introduced the audience to tools, strategies, core skills, processes, and principles which can increase the chances of sustaining change in the life behaviours of their patients.

## Lynn Adams award

The Lynn Adams award is to be bestowed upon those who innovate, inspire, and pioneer in the field of cancer nursing. This year’s recipient was Louisa Fleure, advanced prostate cancer clinical nurse specialist at Guy’s and St Thomas’ NHS Trust, for her paper on ‘Living Healthy on Hormones: A service development to improve men’s experience and knowledge of androgen deprivation therapy (ADT) and its side effects’.

## Poster awards

With more than 100 posters being submitted and presented, the task of selecting the top three was a difficult one indeed. The following posters received awards:

- First place: Allison Irwin from Belfast Health and Social Care Trust for ‘A review of skin reactions in patients with anal cancer receiving concurrent chemotherapy/radiotherapy’.
- Second place: Carrie Flanagan from the University of Ulster/Belfast Health and Social Care Trust for ‘Decision aid for reconstructive surgery following mastectomy: Option Grid’.
- Third place: Renee Reid from Belfast Health and Social Care Trust for ‘The development of a Teenage and Young Adult Cancer Service team’.

## President’s award

To mark the end of their period of office, each outgoing President is invited to present an award to someone who has made an outstanding contribution to UKONS over the previous two years. Natalie Doyle elected to present the award to Ben Hartley, Project Manager at The Royal Marsden, NHS Foundation Trust.

## Conference close

The 2014 UKONS Annual Conference addressed three major themes which are of abiding importance to cancer nursing. These were ‘Living With and Beyond Cancer’, ‘Patient Information and Support’, and ‘Innovations in Treatment and Care’. Attendees of the 2014 UKONS Annual Conference benefitted from the enlightening presentations, interactive workshops, varied exhibitions, and lively poster discussions.

Next year’s UKONS Annual Conference will be held in Birmingham in November 2015. Details of the event will be posted on the UKONS website when available.
